# Gap junctional communication in health and disease

**DOI:** 10.3389/fphys.2014.00442

**Published:** 2014-11-21

**Authors:** Georg Zoidl, David C. Spray

**Affiliations:** ^1^Department of Psychology, York UniversityToronto, ON, Canada; ^2^Dominick P. Purpura Department of Neuroscience, Albert Einstein College of Medicine of Yeshiva UniversityNew York, NY, USA

**Keywords:** connexin, pannexin, neurotoxicity, migrane, epilepsy, inflammation, regeneration, cytoskeleton

Gap junctions (GJ) provide intercellular communication in all multicellular animal species, being as evolutionary distant as worm and men. In the traditional view GJs are known as specialized cell junctions forming communicating channels connecting the cytoplasm of adjacent cells. In almost all tissues GJ coupled cells can rapidly exchange ions, nutrients, or small metabolites to establish electrical coupling or balancing metabolites. During the last decade our knowledge about GJ communications (GJCs) has expanded. Today, the connexins and the second family of GJ-type proteins, the pannexins (Panx) are known to form functional gap junctions (Cx), (hemi)channels (Cx), and channels (Panx) permitting direct communication between cells and between cells and the extracellular environment (Sohl et al., 2005; Sosinsky et al., 2011). Not surprisingly, dysfunctional GJC has been incontrovertibly linked to human disease and the number of genetic alterations found in deafness, skin disease, peripheral and central neuropathies, cataracts, and cardiovascular dysfunctions is rapidly increasing (Zoidl and Dermietzel, 2010). This provides an unequivocal demonstration that GJC is crucial for diverse physiological processes throughout the human body. The focus of this review topic is on delineating the most recent developments and technological advancements in the GJ field, highlighting the fast pacing discovery of dysfunctional GJC in congenital and acquired human disease.

In “The Pannexins: Past and Present,” Bond and Naus review the history of pannexin research, retelling the story of the discovery of this phylogenetically older gene family that forms gap junctions in invertebrates and describing the advances made toward understanding how they influence physiology in both health and disease (Bond and Naus, 2014).

The review “Gap junctions in *C. elegans*,” by Simonsen, Moerman, and Naus, highlights innexins, the gap junction proteins found in invertebrates. They are presented in the context of offering insights into various functions of these intercellular channels in a simple animal model that offers great potential for genome wide functional studies (Simonsen et al., 2014).

Boyce, Wicki-Stordeur, and Swayne review on “Powerful partnership: crosstalk between pannexin 1 and the cytoskeleton” providing insight how Panx1 trafficking and function are linked to the cytoskeleton, a multi-component network that provides critical structural support, transportation, and scaffolding functions in all cell types (Boyce et al., 2014).

Understanding biophysical properties of connexin and pannexin channels is highly relevant in the context of disease pathophysiology and drug development. Xin and Bai review “Functional roles of the amino terminal domain in determining biophysical properties of Cx50 gap junction channels,” providing mechanistic insight on the roles of a particular domain of the Cx50 gap junction channel, the major lens connexin, playing an important role in the development of eye cataracts (Xin and Bai, 2013).

Kurtenbach, Kurtenbach, and Zoidl address the “Relevance of gap junction communication (GJC) for heart function.” To determine the role of GJC in heart disease, they advocate for exploring the gap junction network (GJN) using large scale data mining of publicly available data sets from experimental and (pre)clinical studies (Kurtenbach et al., 2014).

“The role of pannexin hemichannels in inflammation and regeneration” by Makarenkova and Shestopalov provides an overview how post-injury inflammatory response and the subsequent process of tissue regeneration involve cell-cell communication. They conclude that connexins and pannexins play important roles in these processes and should be considered as novel therapeutic targets (Makarenkova and Shestopalov, 2014).

“Molecular pathways of pannexin1-mediated neurotoxicity” by Shestopalov and Slepak, reviews how Panx1 is involved in neurotoxic pathways that control paracrine signaling and regulation of the inflammasome. The most current experimental evidence supports a model where prolonged opening of Panx1 channel induced by danger signals trigger a cascade of neurotoxic events capable of killing retinal ganglion cells (Shestopalov and Slepak, 2014).

The review “Influence of drugs on gap junctions in glioma cell lines and primary astrocytes *in vitro*” by Moinfar, Dambach, and Faustmann summarizes recent advances on our understanding of GJC in different brain cells as well as in gliomas (Moinfar et al., 2014).

The “Roles of gap junctions, connexins, and pannexins in epilepsy” is reviewed by Mylvaganam, Ramani, Krawczyk, and Carlen. They summarize how modulation of gap junctional communication and altered connexin expression are intimately involved in the processes of epilepsy and seizures. Since specific roles in this condition are still not fully understood, they advocate for more specific modulating agents holding the promise of better defining how these proteins modulate and are modulated by seizures, both as gap junctions and membrane channels (Mylvaganam et al., 2014).

In “Migraine with aura” a disabling primary disorder in which patients experience visual, sensitive, or motor symptoms preceding the headache is described. Sarrouilhe, Dejean, and Mesnil present an overview of the involvement of gap junction channels between neurons and glial cells in the neocortex and the trigeminal ganglion in the pathophysiology of this disease, providing an outlook on how gap junction channels have emerged as a potential target in prophylaxis treatment of migraine with aura (Sarrouilhe et al., 2014).

In the article “Relevance of gap junctions and large pore channels in traumatic brain injury” by Nora Prochnow, she addresses the outstanding role of GJC in the pathophysiology of TBI and links the current state of results to applied clinical procedures as well as perspectives in acute and long-term treatment options (Prochnow, 2014).

In closing, Meier and Rosenkranz focus on the modulation of “Cx43 expression observed after transplantation of stem cells in animal models of traumatic and hypoxic brain injury.” The authors develop the intriguing hypothesis that these changes in gap junctional communication might provide one possible explanation for the beneficial effects associated with exogenous stem cell application (Meier and Rosenkranz, 2014). Underlying mechanisms, including the direct communication between host cells and transplanted cells, are discussed.

The connexin/pannexin field is now at the point where there are numerous known hereditary connexin diseases, most of which are believed to be due to gap junction dysfunction/expression disturbance (Laird, 2014), although for some hemichannel involvement has been claimed or speculated (Levit et al., 2012). There are as yet no demonstrated pannexin diseases (Dahl and Keane, 2012; Penuela et al., 2014; Velasquez and Eugenin, 2014), although pannexin null mice exhibit abnormal behavioral phenotypes and response to brain injury (Santiago et al., 2011; Bargiotas et al., 2012; Gulbransen et al., 2012; Prochnow et al., 2012; Lutz et al., 2013; Negoro et al., 2013), suggesting that brain disease or vulnerability to insult might arise from or gets accelerated by mutations in pannexin genes.

Protein complexes formed by specific connexins and pannexins in specific cell types under specific conditions have not yet been extensively enough studied to enable definitive evaluation of the roles of each of the gap junction proteins in various pathological conditions. Studies such as those described here are beginning to scratch the surface of this need, but are critical for development of specific pharmacological and other therapeutic tools to selectively manipulate gap junction function under physiological and pathological conditions.

## Conflict of interest statement

The authors declare that the research was conducted in the absence of any commercial or financial relationships that could be construed as a potential conflict of interest.
